# Enhancing access to contraception through pharmacist prescribing across Canada

**DOI:** 10.1177/17151635211034534

**Published:** 2021-09-13

**Authors:** Judith A. Soon, Anne Marie Whelan, Nesé Yuksel, Sally Rafie

**Affiliations:** Faculty of Pharmaceutical Sciences, University of British Columbia; Department of Family Practice, University of British Columbia; College of Pharmacy, Dalhousie University, Halifax, Nova Scotia; Faculty of Pharmacy and Pharmaceutical Sciences, University of Alberta, Edmonton, Alberta; Skaggs School of Pharmacy and Pharmaceutical Sciences, University of California–San Diego, California

## Introduction

Over the past 20 years, pharmacists across Canada have gradually become more proactive and involved in the continuum of care for reproductive health. Beginning in 2000, pharmacists were able to prescribe emergency contraception in British Columbia, and this gradually spread across the country.^1^ Recently, pharmacists have become pivotal in dispensing and counselling related to medications for medical abortion.^2^ The time is now for pharmacists across Canada to further expand their professional responsibility to be able to prescribe a range of contraception to better meet the needs of their patients.* This is consistent with the goals of the multiyear Canadian Pharmacists’ Harmonized Scope 2020 national initiative.^3^ A key domain of the Harmonized Scope is Prescriptive Authority, with a focus on facilitating consistent full scope of prescribing authority for pharmacists across the country to enhance timely access to health care services.

Convenient and dependable access to prescription contraception across Canada can be challenging, regardless of geographic location.^4^ As many patients do not have a family physician, time-consuming delays in accessing a prescriber for birth control can inadvertently contribute to unintended pregnancies, with potentially onerous personal and societal consequences.^5^ Research suggests that many patients visit their primary care pharmacists more frequently than their primary care physician.^6^ Enabling community-based, primary care pharmacists to practise to their full scope of practice has the potential to facilitate proactive and comprehensive interventions to time-sensitive prescribed medications such as contraception.^7-9^

## Contraception need, usage and access

Statistics Canada reports that 38.5% of females in Canada in 2019 were in the reproductive age range of 15 to 44 years.^10^ Although there are effective contraception choices available in Canada, the products are not always being used effectively or at all in some instances, leading to unintended pregnancies and subsequent abortions.

The 2016 United Nations Human Rights Commissioner’s report of the Committee on the Elimination of Discrimination Against Women specifically addressed the unmet need for contraception in Canada. The report noted that there was a “lack of a comprehensive set of national guidelines or standards for education on sexual and reproductive health and rights curriculum, which resulted in severe discrepancies among provinces/territories in terms of curricula.”^11^ The United Nations Committee also expressed their concern with disparities in access to affordable contraceptives and recommended that Canada “make affordable contraceptives accessible and available to all women and girls, in particular those living in poverty and/or in remote areas.”^11^

The exact percentage of unintended pregnancies in Canada is unknown but was estimated in 2015 to be approximately 40%.^12^ Patients in remote and rural areas frequently experience barriers in accessing abortion services, as a decline in hospital provision may require patients to travel distances to obtain medical abortion services in more urban settings.^13^

There are compelling financial reasons to address the reported underutilization of contraception across Canada through harmonization of contraception prescribing by pharmacists across the country. Each year in Canada, there are estimated to be more than 180,700 unintended pregnancies, often due to inconsistent contraceptive adherence, with an estimated direct cost of over $320 million annually.^12^ In British Columbia, a cost-benefit analysis conducted by Options for Sexual Health in 2010 estimated the provincial government could save $95 million/year if it covered the costs of universal access to prescription contraception.^14^

The Society of Obstetricians and Gynaecologists of Canada’s most recently published clinical contraception consensus guidelines recognize the contraception needs of Canadian women and state that “it is feasible and safe for contraceptives and family planning services to be provided by appropriately trained allied health professionals such as midwives, registered nurses, nurse practitioners and pharmacists” and to recommend that the scope of practice for these professionals should be expanded to promote task sharing in this area.^15^ In the winter of 2020, in response to concerns about reproductive rights during the coronavirus disease 2019 (COVID-19) pandemic, Action Canada for Sexual Health & Rights wrote a letter to the Canadian Minister of Health, urging, among other actions, that the government engage all jurisdictions in Canada to let pharmacists prescribe contraception.^16^ These statements support data from the World Health Organization that there is an unmet need for family planning and contraception. The World Health Organization also suggests an approach to manage this by supporting allied health professionals, including pharmacists, to fill this gap in care.^17^ Pharmacists have the necessary training and the established patient rapport to play a pivotal role in addressing the contraception needs of reproductive-aged patients in Canada.

In January 2020, the Canadian Pharmacists Association (CPhA) conducted an online survey of a random sample of 1500 adult women and men related to accessing treatment for women’s health issues, weighted by census data to correspond to the population in Canada.^18^ Among women with experience using birth control, 72% felt that enabling pharmacists to be actively engaged with screening, prescribing, counselling and managing ongoing contraceptive therapy would result in better access to birth control. Results of the survey are consistent with a previous CPhA patient survey in 2017, which recognized the important role that pharmacists have within the health care system and that patients welcomed the further expansion of pharmacy services beyond filling prescriptions.^19^

There are many countries and jurisdictions around the world, including some areas in the United States (see section below, “Lessons learned from the United States”), where pharmacists have more direct involvement in the provision of contraception. There is plentiful evidence supporting the role of pharmacists in task sharing for contraception care, since people are able to self-screen, and no tests are required, with the exception of a blood pressure measurement for methods containing estrogen.^20^

## Current status of contraception prescribing in Canada

Canadian pharmacists are knowledgeable, accessible and trusted health care professionals whom patients support to provide innovative and expanding professional services related to medication expertise, timely access and convenient locations. As front-line primary health care providers, pharmacists routinely provide customized patient-specific medication information, as well as monitor for potential drug interactions and allergies using up-to-date information on provincial administrative databases.

The 2020 CPhA graphic, “Community Pharmacists in Canada: Contraceptive Prescribing,” provides a map of the 4 provinces (Alberta, Saskatchewan, Quebec, Nova Scotia) where pharmacists are able to prescribe hormonal contraception and highlights current gaps in access to birth control in Canada.^21^ In these 4 jurisdictions, pharmacists are able to prescribe not only birth control pills but also hormonal contraceptives that do not require daily administration (e.g., vaginal rings, transdermal patches). Medroxyprogesterone acetate, a progestin-containing injection, can also be prescribed and administered every 3 months. Long-acting reversible contraception (LARC), such as an intrauterine device (copper), intrauterine system (progestogen) or the newly released etonogestrel implant, has the potential to be prescribed by a pharmacist when in an arrangement with a primary care physician able to schedule an appointment for insertion (Table 1). While hormonal contraceptives are on provincial formularies and covered by third-party insurers, individual patient coverage and plans will vary.

**Table 1:** Pharmacists’ expanded scope of practice for prescribing to initiate contraception*

Prescriptive authorization	Method of contraception	BC	AB	SK	MN	ON	QC	NB	NS	PE	NL	YT	NT	NU
No current authorization in place^1^	Unable to prescribe contraception	X			X	X		X		X	X	X	X	X
**Additional prescribing authorization^2^**	*Alberta*: Oral, transdermal patch or vaginal ring hormonal contraception, progestin only injection^3^, progestin intrauterine contraception6, etonogestrel subdermal implant.^3^	X	✓	X	X	X	X	X	X	X	X	X	X	X
**Minor ailments**	*Saskatchewan*: Oral, transdermal patch or vaginal ring hormonal contraception, progestin only injection^2^, etonogestrel subdermal implant.^4^	X	X	✓	X	X	X	X	X	X	X	X	X	X
**Prescribing preventative medicines**	*Quebec*: Oral, transdermal patch or vaginal ring hormonal contraception, progestin only injection.	X	X	X	X	X	✓	X	X	X	X	X	X	X
**Prescribing preventative medicines**	*Nova Scotia*: Oral, transdermal patch or vaginal ring hormonal contraception, progestin only injection^2^, progestin intrauterine contraception^5^, etonogestrel subdermal implant.^4^	X	X	X	X	X	X	X	✓	X	X	X	X	X

1 Bold X signifies an opportunity to enhance access to contraception through pharmacist prescribing. BC British Columbia; AB Alberta; SK Saskatchewan; MN Manitoba; ON Ontario; QC Quebec; NB New Brunswick; NS Nova Scotia; PE Prince Edward Island; NL Newfoundland; YT Yukon; NT Northwest Territories; NU Nunavut.

2 2Pharmacists can administer the injection; 3Arrange for insertion with primary care provider (physician/nurse practitioner); 4Implant status to be determined, 5In arrangement with primary care prescriber (physician/nurse practitioner).

* Note: in many provinces/territories, pharmacists can prescribe to adapt/manage contraceptives to make therapeutic substitution, change drug dosage/formulation/regimen or renew/extend prescriptions for continuity of care. CPhA Pharmacists’ Expanded Scope of Practice. https://www.pharmacists.ca/pharmacy-in-canada/scope-of-practice-canada/ Accessed November 15, 2020.

**Table:** Box 1 Recommendations

*1. Harmonize prescriptive authority for contraception across Canada* Facilitating consistent full scope of prescribing authority across Canada is a focus of the recently launched Canadian Pharmacists’ Harmonized Scope 2020 initiative. The initiative promises to “support and enable national and provincial efforts to extend, expand and harmonize scope of practice across the country.”^3^ Trusting provincial relationships developed through the current CPhA Pharmacists’ Opioid Stewardship initiative may facilitate the recognition of pharmacists as collaborative health care professionals and enable similar harmonization strategies for contraceptives. *2. Create technical assistance tools for pharmacy-based contraception care* A well-accepted pharmacist checklist and resource guide have recently been developed for the pharmacist dispensing of mifepristone/misoprostol for medical abortion.^60^ Similar resources for contraceptive prescribing would be a valuable asset to enable the standardization of contraceptive prescribing across the country, among other tools. *3. Develop a curriculum framework for contraception* The curriculum framework would include independent prescribing for contraception that would align in all schools of pharmacy and continuing professional development (CCCEP-accredited) programs for practising pharmacists. *4. Standardize pharmacist remuneration payment for contraception care visits* A sustainable infrastructure that enables payment for the initial assessment, prescribing and follow-up visits by pharmacists must be implemented in all jurisdictions to optimize the implementation of contraception care by pharmacists. *5. Conduct pharmacy practice research* Conduct research to facilitate implementation, evaluate the program and measure outcomes of pharmacist contraceptive prescribing.^51^ Implementation science studies should research pharmacist barriers and facilitators, system-based barriers/facilitators and patient elements. Program evaluations should describe patient utilization, satisfaction and patterns of contraceptive care. Outcomes studies will measure reach and impact.

## Contraception curriculum and training in Canada

Studies from the United States have indicated that pharmacists desire additional training related to prescribing hormonal contraception.^22,23^ Pharmacists in Canada have also indicated the need for additional training to take on emerging roles, such as prescribing.^24^ In a survey from Alberta, pharmacists identified additional training in physical assessment, interpreting laboratory tests and making drug therapy decisions required to take on these roles.^25^ There is little research looking at the specific training needs of Canadian pharmacists with regards to contraception prescribing. In a British Columbia initiative, pharmacists indicated they had the skills for assessment, decision-making and follow-up for contraception task-sharing.^4^ During the interviews, pharmacists mentioned that they would appreciate specific training to update their contraception knowledge.^4^

The World Health Organization competencies in sexual and reproductive health outline the importance of health professional training on contraception.^26^ A strong education starting in the undergraduate program sets the foundation for future practitioners for lifelong learning. Students need to have the knowledge, skills *and* the confidence to prescribe contraception by the time they graduate. Components of contraception knowledge include contraceptive options, contraindications, adherence considerations, prioritizing options and management of side effects. Skills for prescribing include history-taking, patient assessment, patient education/counselling, decision-making, follow-up, how to adjust therapy and knowing when to refer.

It is also essential that Canadian undergraduate pharmacy curriculum provides opportunities for learning effective decision-making and problem-solving skills to build prescribing confidence.^27-30^ What is known about educating students for contraception prescribing? Pharmacy students expressed a high level of confidence with contraceptive counselling skills but highlighted the need for more education in choosing contraception options.^31^ Health care simulation has been shown to improve pharmacy students’ self-perceived knowledge and confidence.^32^ A connection between curriculum activities and increased comfort and skills with provision of emergency contraception has been reported, which may translate to contraception prescribing.^33^ Education should incorporate components of active learning strategies and skill development to build student confidence. These opportunities should be embedded throughout the curriculum, with multiple scenarios for students to practise.

In a survey on the contraception curricula in US pharmacy schools, a mean of 6.8 hours was dedicated to contraception, with much variability between programs in hours and teaching delivery.^34^ The authors indicated that the criteria for what are adequate hours and content for contraception curriculum are unknown.^34^ In a recent survey of sexual and reproductive health content in Canadian pharmacy undergraduate programs, the hours reported for contraception were even higher, with the majority of programs indicating over 10 hours (N. Yuksel, unpublished data). Reassuringly, most Canadian programs are now addressing contraception prescribing, with pharmacist prescribing commonly identified as the major change to the sexual and reproductive health curriculum in the past 5 years.

Undergraduate curricula and continuing professional development for pharmacists should be aligned. In the United States, standardized training programs for pharmacist prescribing of contraception vary between states, ranging from 2 to 6 hours of mandatory training.^34^ In Canada, while most jurisdictions with contraception prescribing have not mandated additional training, some provinces have training requirements to prescribe for ambulatory conditions.^35^ Jurisdiction-specific continuing education opportunities for prescribing contraception are available across Canada.^36^ CPhA is committed to women’s health initiatives, including providing website resources and delivering webinars on reproductive health.^36^

Multiple formats for continuing professional development would help target different learners. Preferred formats for contraception training identified by pharmacists include short live sessions, on-demand webinars and online self-study programs.^22^ Pharmacists have also expressed that social aspects, such as learning with peers, interprofessional teams or in the workplace, are important to their professional development.^24^ Additional tools may be needed to support pharmacists in practice such as risk assessment questionnaires, practice tools or procedural algorithms or protocols.^4,22^ Some provinces such as Saskatchewan and Nova Scotia have provided supplementary guidelines, algorithms and documentation tools for assessment and prescribing.^36,37^

## Lessons learned from the United States

Direct access to contraception in community pharmacies is being quickly adopted across the United States as a strategy to address challenges with access to contraception. The mechanism for pharmacists to prescribe contraception is at the state level and can be accomplished under statewide protocol, standing order or collaborative practice agreement, also known as collaborative drug therapy agreement.^38^

Beginning in 2016, specific contraception prescribing authorities have been granted by legislation in 18 states (California, Oregon, Colorado, Hawaii, Maryland, Utah, New Mexico, Tennessee, West Virginia, Virginia, Minnesota, New Hampshire, Vermont, Arkansas, Arizona, Delaware, Illinois, Nevada, plus Washington, DC) have used collaborative practice agreement opportunities to provide pharmacy access to contraception.^39^ In these states, pharmacists can prescribe contraception after completing a training program. When visiting a pharmacy for contraception, the patient meets with a pharmacist for screening, assessment and counselling before receiving a prescription and same-day supplies.^40^

The pharmacist’s role in contraception care continues to take hold in the United States as more states consider, pass and implement policies expanding pharmacist prescribing of contraception. These efforts build on prior state efforts to increase direct access to emergency contraception in pharmacies, as well as other pharmacy-based public health interventions such as immunizations, tobacco cessation and naloxone. Pharmacist-prescribed contraception is expected to become the standard of practice in community pharmacy settings in most, if not all, states in the near future.

Interest from Canadians is consistent with reports from women in the United States, who have expressed interest in direct pharmacy access to contraception and have rapidly used hormonal contraception prescribing services once available.^41-44^ Other health care providers and major medical associations have supported this model of access.^45-47^ Pharmacists and student pharmacists are strongly interested in participating in contraception care, increasing direct patient care services and facilitating patient access to a public health intervention.^21,48-53^

There are now over 3500 pharmacies in 18 states offering pharmacy access to contraception, although this is less widespread than desired.^54^ For example, while only 5% to 11% of community pharmacies were offering pharmacist-prescribed contraception in California after 1 year, that increased to approximately 25% after 4 years.^55,56^

While the state policies facilitate implementation of this service, numerous barriers mitigate the potential reach and effectiveness of these initiatives. Implementation level varies by state, largely due to structural barriers, such as patient age restrictions or limited training options, and facilitators, such as payment for pharmacist services.^57^ Lack of payment for pharmacist services has emerged as the biggest structural barrier.^38^ Birth Control Pharmacist (https://birthcontrolpharmacist.com/) serves as a coordinating centre for tracking policies, advocacy, dissemination and implementation, including training and practice tools, public awareness and research.

A multistate evaluation of pharmacist prescribing of contraception found provision of a greater supply of medication compared to traditional clinicians, potentially improving contraceptive continuation.^58^ Early findings suggest that pharmacist prescribing of hormonal contraception has a positive impact on unintended pregnancies and health care costs.^59^

## Conclusions

Pharmacist prescribing has been shown to enhance contraceptive access for people of all ages and provide convenient and timely access to care. To facilitate implementation of this crucial service and enable pharmacists to practise to their full scope, pharmacist prescribing of hormonal contraceptives should be harmonized across Canadian jurisdictions. Contraception education should be standardized in pharmacy school curricula, and technical support tools should be developed and made readily available. This policy initiative to enable pharmacists to prescribe hormonal contraceptives has the potential to not only improve access but also increase the overall utilization of highly effective contraception across the country. ■

## Footnotes

* Throughout this paper, we use gender-inclusive language to refer to reproductive health and services for all people who may benefit from them. When describing studies, the samples are represented as originally described.
